# Comparative metagenomic analysis of bacterial and fungal communities associated with bayoud-resistant and susceptible date palm cultivars in the Zagora oasis-Morocco

**DOI:** 10.1186/s12866-026-04837-8

**Published:** 2026-02-23

**Authors:** Aliou Moussa Diouf, Abdou Lahat Mbaye, Maimouna Deh, Rachid Lahlali, Mohamed Aziz Elhoumaizi, Zineb Rchiad, Mustapha Barakate

**Affiliations:** 1 Agrobiosciences Department, College of Agriculture and Environmental Sciences, Mohammed VI Polytechnic University (UM6P), Ben Guerir, 43150 Morocco; 2 Phytopathology Unit, Department of Plant Protection, Ecole Nationale d’Agriculture de Meknès, BP S/40, Km10, Rte Haj Kaddour, 50001 Meknes, Morocco; 3 Laboratory for Agricultural Productions Improvement, Biotechnology and Environment (LAPABE), Faculty of Sciences, University Mohammed First, BP:717, Oujda, 60000 Morocco; 4 Biosciences CoreLab, Mohammed VI Polytechnic University (UM6P), Ben Guerir, Morocco; 5 Faculty of Sciences-Semlalia, Laboratory of Water Sciences, Microbial Biotechnologies, and Natural Resources Sustainability (AQUABIOTECH), Unit of Microbial Biotechnologies, Agrosciences, and Environment (BIOMAGE)-CNRST Labeled Research Unit N°4, Cadi Ayyad University (UCA), PO Box 2390, Marrakesh, 40000 Morocco

**Keywords:** *Fusarium oxysporum* f. sp. *albedinis*, Microbial metagenomics, Biological control, Bacteria, Fungi,, Co-occurrence network

## Abstract

**Background:**

*Fusarium oxysporum* f. sp. *albedinis* (Foa) is a destructive soil-borne fungal pathogen responsible for bayoud disease, which threatens date palm cultivation in North Africa. This disease has caused significant agricultural losses, particularly in Morocco, where the Zagora oasis is a key region for date palm production. Within this oasis, two cultivars—Black Bousthammi and Jihel—are mainly cultivated and exhibit complete resistance and high susceptibility to Foa, respectively. Thus, this study aimed to identify and compare the bacterial and fungal communities associated with the two cultivars and understand their assemblage regarding the disease resistance or susceptibility. Moreover, we explored the influence of each cultivar on the composition and structure of its root-associated microbiome and examined its relationship with the microbial populations present in the surrounding bulk soil, to better understand the recruitment dynamics that shape the microbiome in the roots.

**Results:**

The results revealed significant differences in microbiome composition between the bulk soil and roots of the two date palm cultivars, and between the microbiome of the resistant and susceptible cultivars as well. Moreover, we observed that date palm cultivars had a greater effect on bacterial community composition than on fungal population. Interestingly, the susceptible cultivar exhibited a higher enrichment of several beneficial genera, such as *Pseudomonas*, *Lysinibacillus*, *Actinomadura*, *Halomonas*, *Kocuria*, *Serratia*, *Phyllobacterium*,* Bacillus*,* Streptomyces*, and *Trichoderma*.

**Conclusion:**

The presence of these beneficial genera, known for their antagonistic activity against phytopathogens, may reflect a recruitment pattern associated with pathogen pressure in the susceptible cultivar. This study is the first to compare the microbial communities between a bayoud-resistant and susceptible cultivar and provides insights into the potential role of the root microbiome when plants are under pathogen pressure. This reinforces the need to further elucidate the genetic and biological mechanisms that trigger microbiome assembly, which could be a key step in developing effective methods to manage the bayoud disease.

**Supplementary Information:**

The online version contains supplementary material available at 10.1186/s12866-026-04837-8.

## Background

Plants recruit root-associated microbes from the soil, which is a reservoir of microorganisms. This recruitment is particularly influenced by plant root exudates which selectively enrich the rhizosphere with selected groups of microorganisms [1]. Through this process, plants foster a diverse community of beneficial microorganisms that can facilitate nutrient uptake, stimulate growth [2, 3], and play crucial roles in mitigating biotic stress caused by soil-borne pathogens [4–6]. These microbes compete with pathogenic organisms for resources and space, effectively limiting their proliferation and establishment in the rhizo-compartment [7, 8]. Although the soil microbial community is highly influenced by the soil physicochemical properties [9], there is evidence that the plant microbiome is greatly impacted by plant genotype, which constitutes one of the main factors in shaping the microbial community [10–13].

The use of OMICS has considerably facilitated the study of plant-microbe interactions [11, 12]. While genomics and transcriptomics allow us to gain insight into the molecular features and gene expression patterns, other techniques, such as metagenomics and metatranscriptomics, unlock the limitations of studying gene functions and expression at the community level and within the environmental context. Targeted metagenomics have been particularly used to comprehend microbial community assembly and dynamics in plants. It has been applied to decipher the microbial community of different date palm (*Phoenix dactylifera*) cultivars in Tunisia [10, 14, 15], Dubai [16], Saudi Arabia [17, 18], Oman [19, 20], United Arabe Emirates [21] and Qatar [22, 23]. However, the composition of the core and pan microbial populations of Moroccan date palm cultivars is yet to be elucidated [24, 25].

*Fusarium oxysporum* f. sp. *albedinis* (Foa) poses a major threat to date palm cultivation in Morocco. Although several studies have investigated the resistance and susceptibility of Moroccan date palm cultivars to bayoud disease [26], little to no attention has been given to the role of the associated microbiome. Specifically, the composition, structure, and potential protective functions of microbial communities associated with resistant and susceptible cultivars remain largely unexplored. Understanding how microbial assemblages influence disease dynamics could open new avenues for sustainable management strategies, including the development of biocontrol approaches. Therefore, the main objective of this study was to analyze the bacterial and fungal communities associated with two contrasting date palm cultivars and investigate how these microbial populations are recruited from bulk soil. The two cultivars, Black Bousthammi and Jihel, are respectively resistant and susceptible to bayoud disease [26], and are particularly cultivated in the Zagora oasis, which is affected by Foa. This study provides the first evidence of Moroccan date palm root-associated microbiome and suggest that pathogen pressure could induce the assembly of beneficial micro-organisms in susceptible cultivars.

## Methods

### Sample collection and processing

**Fig. 1:**
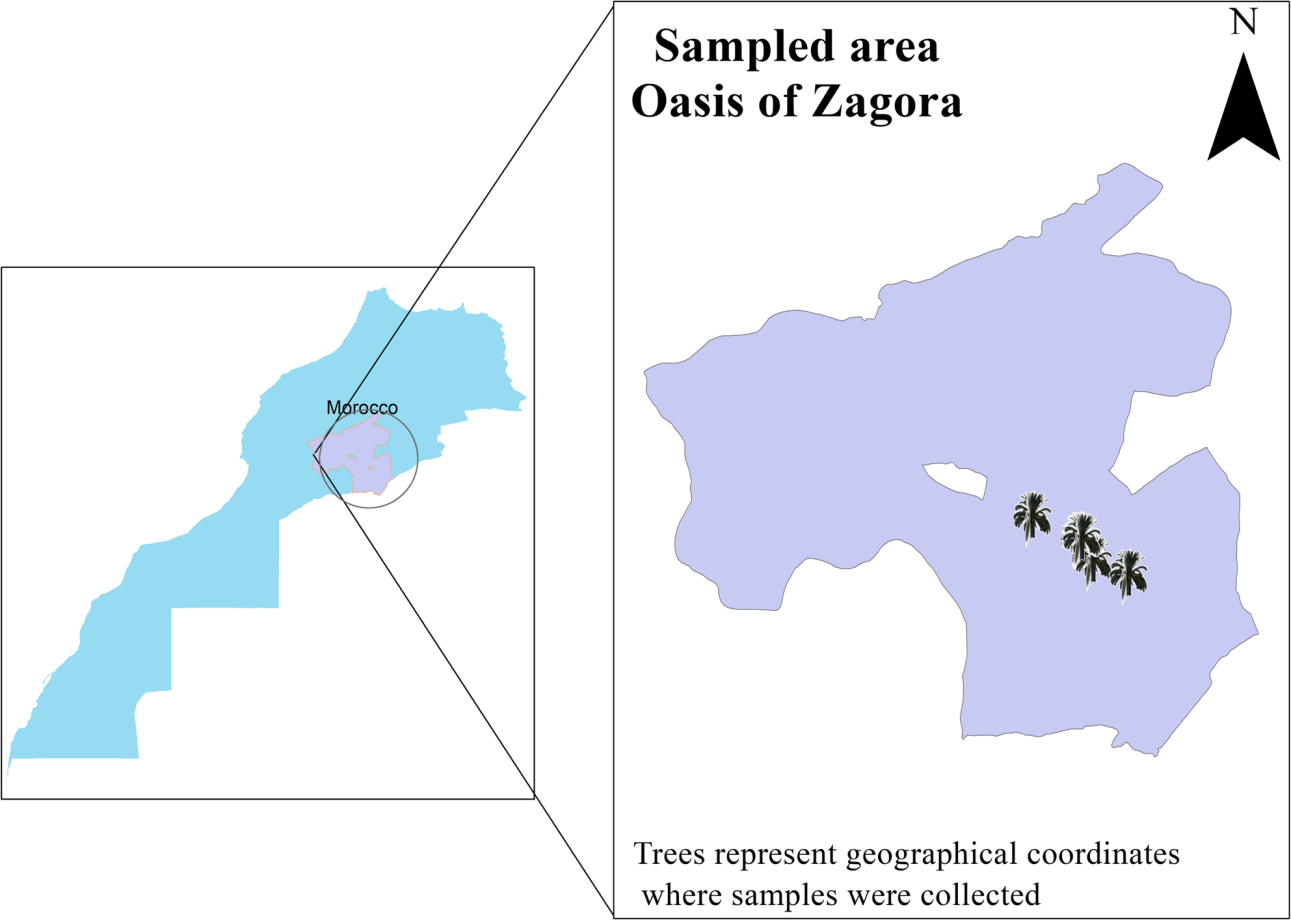
Moroccan map highlighting the location of the Zagora oasis where the samples of the Foa-resistant and susceptible date palm cultivars, Black Bousthammi and Jihel, were collected

Soil samples were collected from Zagora oasis (30°24’56” N, 05°53’39” W), Morocco (Fig. 1), as described by Diouf et al. [27]. Briefly, five biological replicates from the roots and root-surrounding soils of the resistant and susceptible cultivars were collected at 25 cm depth and immediately transported at 4 °C to the laboratory. The samples collected from susceptible cultivars were obtained from trees that showed no visible symptoms of bayoud infection. The samples used for amplicon sequencing were stored at -20 °C until use, while a subset was stored at 4 °C for bacterial isolation. Bulk soil samples were collected from uncultivated soils in the oasis at similar depths. To collect root-associated microbes, the roots were shaken to detach the loosened soil, vortexed for 15s in 25 ml sterile phosphate-buffered saline (PBS, 8 g NaCl; 0.2 g KCl; 1.44 g Na2HPO4 and 0.245 g KH2PO4 /L) and filtered with cheesecloth to remove parts of the plant material. The filtered solution was centrifuged, resuspended in 1 ml PBS, and centrifuged again to collect the soil. Root-surrounding soil was used to study the physicochemical properties of the soil.

### DNA extraction, amplification, and sequencing

Soil DNA was extracted using the FastDNA Spin Kit for Soil (MP Biomedicals) following the manufacturer’s instructions. The V3-V4 region of the 16S rDNA was amplified using the primer pair 5’-CCTAYGGGRBGCASCAG-3’-F and 5’-GGACTACNNGGGTATCTAAT-3’-R, and the ITS1 region was amplified using the primers 5’-CTTGGTCATTTAGAGGAAGTAA-3’-F and 5’-GCTGCGTTCTTCATCGATGC-3’-R. The metabarcoding libraries were prepared following the Illumina protocol. Briefly, barcoding PCR reactions were carried out with 15 µL of Phusion^®^ High-Fidelity PCR Master Mix (New England Biolabs), 0.2 µM of forward and reverse primers, and approximately 10 ng template DNA. Thermal cycling consisted of an initial denaturation at 98 °C for 1 min, followed by 30 cycles of denaturation at 98℃for 10s, annealing at 50 °C for 30 s, and elongation at 72 °C for 30 s and 72 °C for 5 min. Following amplification, PCR products of the proper size were visualized on a 2% agarose gel, and amplicons were purified using magnetic bead purification. After purification, the PCR products from each sample were pooled, end-repaired, A-tailed, and ligated with Illumina adapters. Libraries were validated using Qubit, real-time PCR, and an Agilent 5400 fragment analyser. Quantified libraries were pooled and sequenced on an Illumina sequencer as PE250.

### Amplicon data processing for 16 S and ITS bioinformatic analysis

Data quality was assessed using FastQC [28], and a general report was constructed using the MultiQC package [29]. After controlling the quality of the reads, the data were analyzed using the Qiime2 pipeline [30].To generate a set of representative sequences, low-quality and chimeric sequences were filtered, and paired-end reads were merged with the q2-dada2 plugin implemented in the pipeline to generate Amplicon Sequence Variants (ASVs). To infer bacterial taxonomic annotations for each ASV, we downloaded the Silva database version 138.1 [31]. Low-quality sequences and eukaryotic taxa were removed, and only bacteria and archaea were maintained. After sequence dereplication, the V3-V4 region was extracted using the primer pairs listed above. It has been established that extracting 16 S rRNA sequences using primer sets that amplify the target region increases taxonomic classification [32]. Fungal taxonomy annotation was performed using the UNITE database version 9.0 [33].

### Soil physicochemical properties, statistical analysis of microbial population, and environmental variables

The root surrounding soils of the two date palm cultivars, as well as the bulk soil, were used to assess soil physicochemical properties (Table S2). The downstream analysis of the microbial data was performed using R. First, we investigated the microbial community profile by analyzing the abundance of each bacterial and fungal phylum. Then, we compared the variation within each group of samples by calculating the richness and Shannon index, using the *estimate richness* command implemented in the Phyloseq package [34]. Differences between sample groups were visualized using Principal Coordinate Analysis (PCoA) based on the Bray-Curtis distance. Permutational Multivariate Analysis of Variance (PERMANOVA) was used to compare microbial community composition and verify whether there were significant variations *(p* < 0.05) in community structures. Distance-based redundancy analysis (db-RDA) was used to study the relationships between environmental variables and microbial community composition using the *capscale* function, based on Bray-Curtis dissimilarities. The statistical significance (*p* < 0.05) of each variable was calculated using the *envfit* command, with 999 permutations, implemented in the vegan package [35]. Linear discriminant analysis (LDA) Effect Size (LEfSe) was performed to identify significantly enriched taxa that discriminate between groups. LEfSe, based on the Kruskal-Wallis test (*p* < 0.05) and LDA score cutoff of 2, was performed using the MicrobiomeMarker package [36], and the data was normalized using the CPM method. Co-occurrence network analysis was performed by combining the bacterial and fungal ASVs in a single table, and the counts were transformed to relative abundances. The combined table was filtered based on an abundance threshold of 0.01%, and networks were constructed using the igraph [37] package based on Spearman coefficient correlation (*r* > 0.7) and a significant *p*-value cutoff of 0.01.

### Bacterial isolation, antagonistic assay and detection of Foa in the soil

The roots detached from the loosen soil were shaken for 1 h at 150 rpm in a 250 ml Erlenmeyer flask containing 100 ml of sterile PBS. Serial dilutions were performed on the bacterial suspensions in PBS, plated on Tryptic Soy Broth (TSB), and incubated at 30 °C. Following visual observation, colonies were picked and cultured on new TSB plates. The isolated bacteria were challenged with Foa using the dual-confrontation assay technic to detect antagonistic isolates. The assay was conducted by placing Foa, obtained from an actively growing culture on a Potato Dextrose Agar (PDA) plate, in the center of a petri plate containing PDA. Then, 10 µl of the bacterial suspension, obtained from overnight culture, was placed 2.5 cm away on each side of the fungal plug and incubated at 28 °C for 7 days. After 7 days, the inhibition rate was measured using the following formula:

$$\%\mathrm{Inhibition}=\frac{\left(\text{Control diameter}-\text{Diameter in co-culture}\right)}{\text{Control diameter}}\times100$$

  

The statistical significance of the inhibition rate was computed using IBM SPSS software with a one-way analysis of variance (ANOVA). Significant differences between means were evaluated using Tukey’s multiple comparison test at a *p*-value cutoff of 0.05. The bacteria were identified by sequencing the 16S rRNA, which was amplified using the primer pair 27F (5’-AGAGTTTGATCMTGGCTCAG-3’) and 1492R (5’-GGTTACCTTGTTACGACTT-3’).

Pathogen presence in the soil was detected by extracting DNA, from the root surrounding soil, using the FastDNA Spin Kit for Soil (MP Biomedicals), following the manufacturer’s instructions. The extracted DNA was subjected to PCR, with a control without DNA, using the primer pairs TL3:5’-GATGAAGAGCGAGTAAGTACC-3’ and FOA28:5’-CTTCCACGTCTCTTCTTCTTC-3’, which amplify a specific 400 bp product unique to Foa isolates [38]. Amplified products were visualized on 2% agarose gel.

## Results

### Microbial diversity and community structure analysis reveal statistically significant differences between groups

**Fig. 2:**
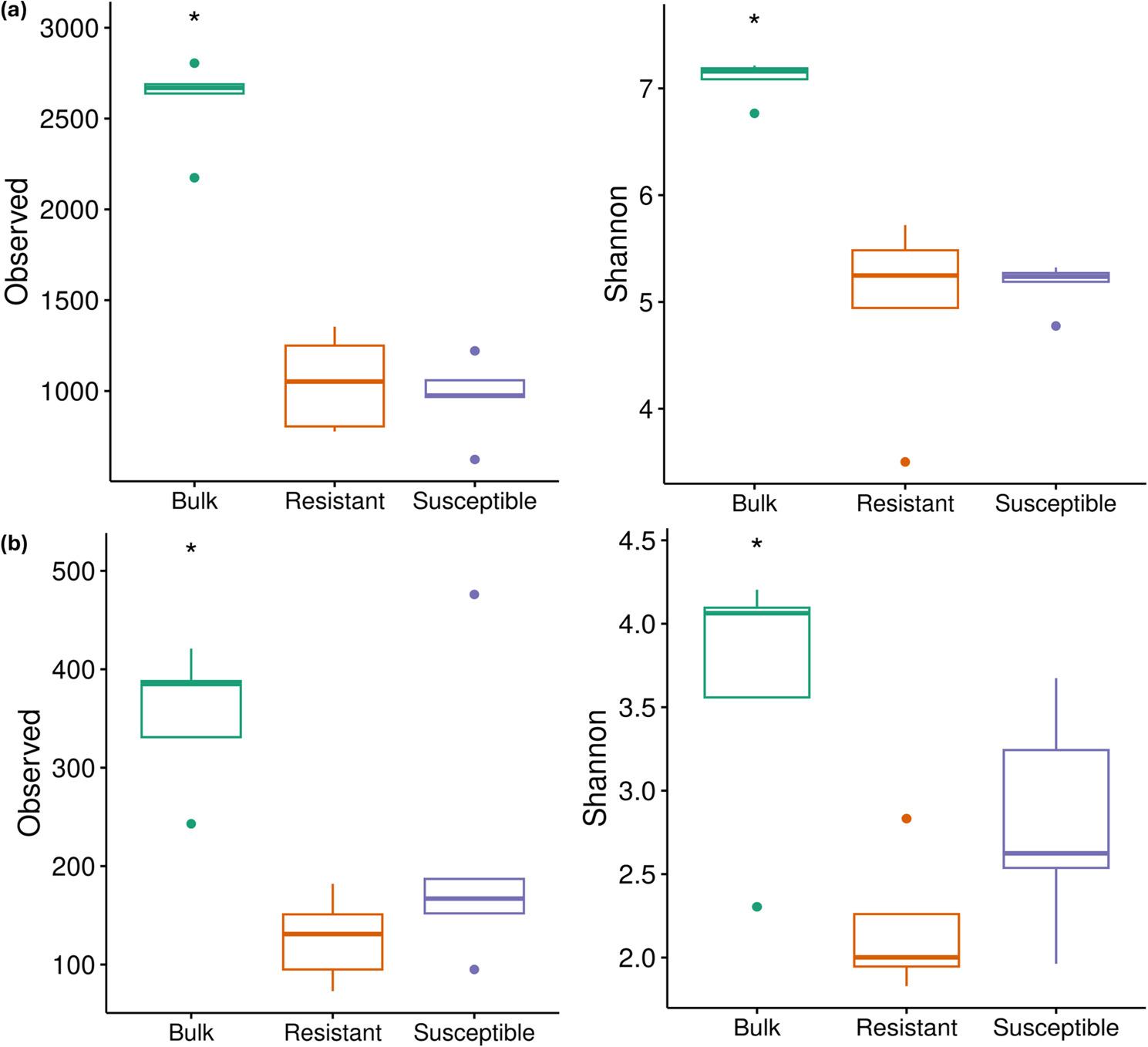
Plot of (**a**) bacterial and (**b**) fungal alpha diversity with Richness and Shannon metrics in bulk, resistant, and susceptible samples. Asterisks (*) represent statistical significance (*p* < 0.05)

After removal of low-quality and chimeric sequences, a total of 970.557 bacterial (the mean number of reads was 64.703 and read numbers varied between 28.434 and 87.188 among samples) and 1.246.637 fungal (the mean number of reads was 83.109, and read numbers varied between 49.060 and 104.498 among samples) high-quality paired-end reads were obtained from the 15 samples. The bacterial sequences were grouped into 12.853 unique ASVs, and the fungal sequences generated 1.958 unique ASVs. Alpha diversity analysis revealed that the diversity within bulk samples was significantly higher than that of resistant and susceptible cultivars for both bacteria and fungi (Fig. 2). The comparison between resistant and susceptible cultivars revealed slightly greater bacterial diversity in the resistant cultivar, whereas the fungal community exhibited higher diversity in the susceptible cultivar. However, these variations were not statistically significant (*p* > 0.05).

**Fig. 3:**
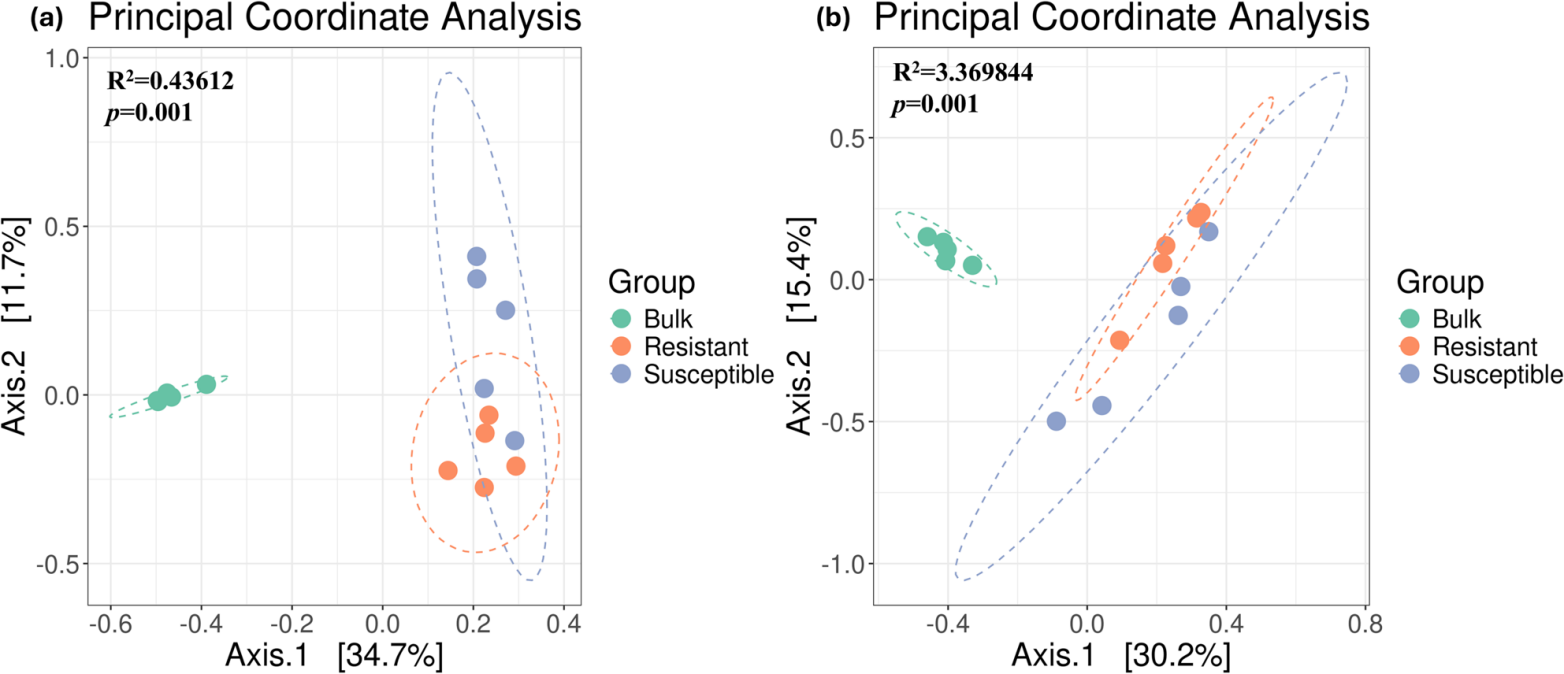
Principal coordinate analysis of (**a**) bacterial and (**b**) fungal communities in bulk, resistant, and susceptible cultivars based on the Bray-Curtis distance

The PCoA computed based on the Bray-Curtis distance explained 46.4% and 45.6% of the variation in the bacterial and fungal communities, respectively (Fig. 3a and b), and showed that bulk samples clustered separately from the resistant and susceptible rhizo-compartment samples. The differences between the susceptible and resistant cultivars were more evident in the bacterial community than in the fungal community, resulting in the formation of two distinct clusters, although some overlaps were observed.

These results were further confirmed by PERMANOVA (Table S1), which showed significant community dissimilarities between the bulk and resistant cultivar (*p* = 0. 005), bulk, and susceptible cultivar (*p* = 0. 009), and susceptible and resistant cultivars (*p* = 0.017) in the bacterial population. Expectedly, db-RDA showed no significant differences *(p* > 0.05) in the soil physicochemical properties (Table S2) of the bulk, resistant and susceptible cultivar samples. In the fungal community, dissimilarities were observed between bulk and the resistant cultivar (*p* = 0.008) and between bulk and the susceptible cultivar (*p* = 0.009), whereas the variation in community structure was not significant between susceptible and resistant cultivars(*p* = 0.148).

### Bacterial and fungal community composition in the bulk and rhizo-compartment samples of resistant and susceptible cultivars

**Fig. 4:**
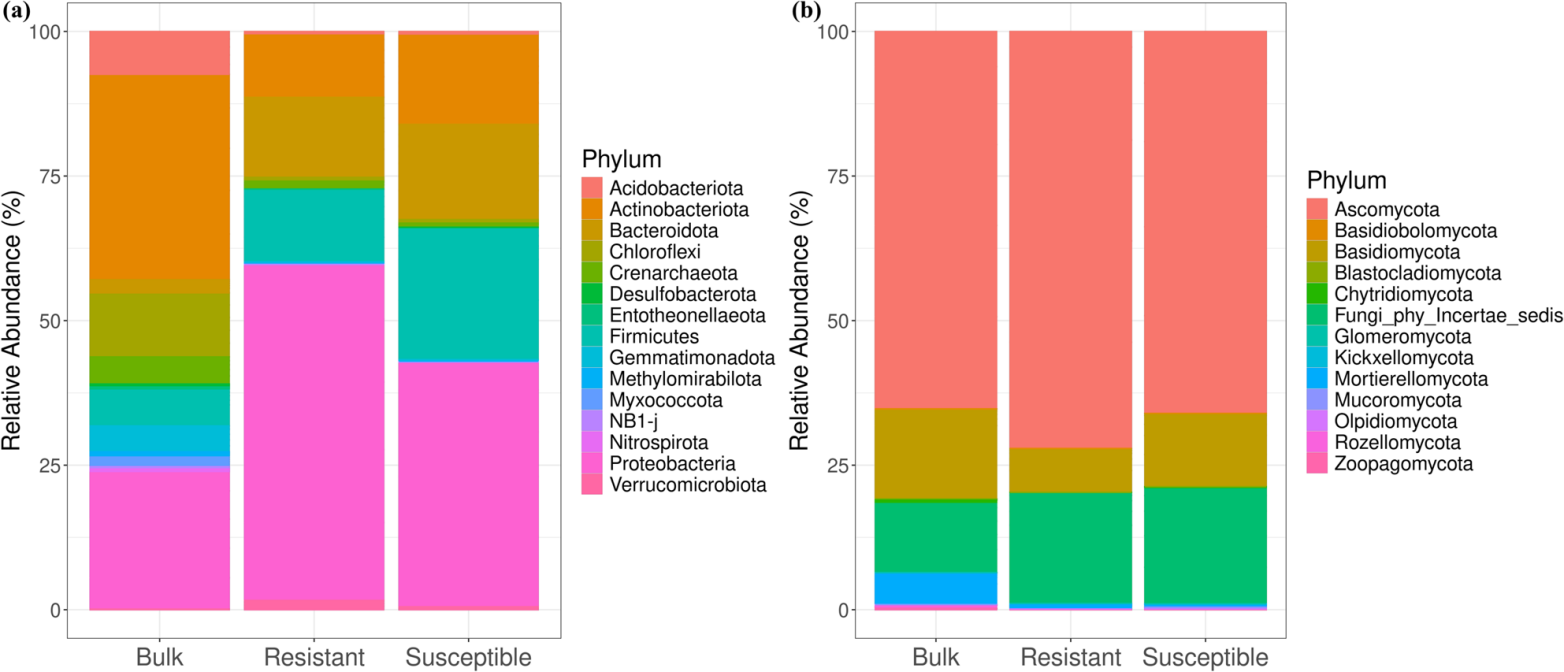
Representation of the top 15 (**a**) bacterial and (**b**) fungal phyla abundance in bulk, resistant, and susceptible cultivars

To decipher the bacterial and fungal communities associated with the collected samples, we investigated the most representative taxa at the phylum level. In the overall composition of the bacterial community, 40 phyla, 110 classes, 224 orders, 433 families, and 931 genera were identified. The most representative phyla (> 1%) were Proteobacteria (41.20%), Actinobacteriota (20.57%), Firmicutes (20.57%), Bacteroidota (10.82%), Chloroflexi (4.08%), Acidobacteriota (2.72%), Crenarchaeota (2.23%), and Gemmatimonadota (1.68%) (Fig. 4a). Bulk soils were dominated (> 1%) by nine bacterial phyla: Actinobacteriota (35.23%), Proteobacteria (23.60%), Chloroflexi (10.96%), Acidobacteriota (7.34%), Firmicutes (6.35%), Crenarchaeota (4.64%), Gemmatimonadota (4.44%), Bacteroidota (2.28%), and Myxococcota (1.87%). The population of Actinobacteriota in the bulk samples was reduced in the roots of date palm, representing 10.92% and 15.57% in the resistant and susceptible cultivars, respectively. The phylum Proteobacteria was dominant in the root compartments, with a higher abundance in the resistant cultivar (57.83%) than in the susceptible one (42.18%). The Firmicutes population was also more represented in date palm roots than in the bulk soil samples, with a greater presence in the susceptible cultivar (22.72%) than in the resistant one (12.45%). The Bacteroidota phylum, which had only 2.2% representation in bulk samples, was consequently enriched in susceptible (16.40%) and resistant (13.77%) cultivars.

Analysis of the fungal community identified 13 phyla, 45 classes, 88 orders, 187 families, and 343 genera (Fig. 4b). Among these phyla, Ascomycota emerged as the dominant group, accounting for 67.81% of the total abundance. Other notable phyla included Fungi_phy_Incertae_sedis (17.16%), Basidiomycota (11.91%), and Mortierellomycota (2.29%). In bulk soils, Ascomycota remained the predominant phylum, comprising 65.26% of the community composition. It was also noticeably abundant in the resistant and susceptible cultivars, with respective proportions of 72.11% and 66.04%. In contrast, Basidiomycota showed a higher representation in bulk soils, comprising 15.53% compared to susceptible (12.65%) and resistant (7.55%) cultivars. This differential representation highlights the varying dynamics of fungal communities within different soil contexts and among plant cultivars. 

**Fig. 5:**
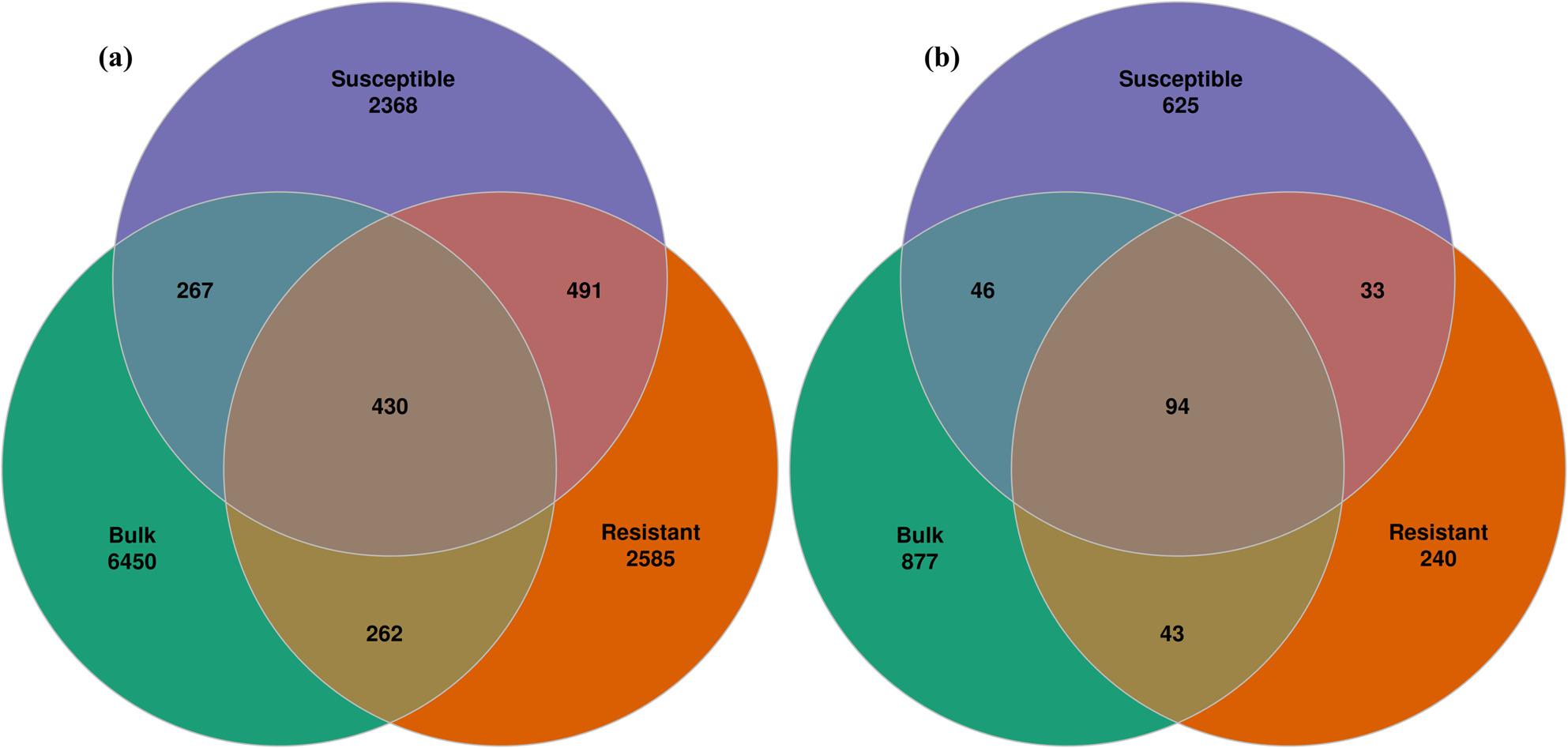
Venn diagram showing unique and shared (**a**) bacterial and (**b**) fungal ASV between bulk, resistant, and susceptible cultivars

The investigation of unique and shared ASVs revealed a core of 430 and 94 ASVs in the bacterial and fungal communities, respectively (Fig. 5). In the bacterial community, the susceptible cultivar shared 267 ASVs with bulk samples and 491 ASVs with the resistant cultivar, whereas 262 ASVs were shared between bulk and resistant cultivar samples. A total of 2368 ASVs were unique to the susceptible cultivar, whereas 6450 and 2585 ASVs were unique to bulk and resistant cultivar samples, respectively. Fungal analysis showed 625 unique ASVs in the susceptible cultivar, 240 in the resistant one, and 877 in bulk samples. Bulk samples shared 46 and 43 fungal ASVs with susceptible and resistant cultivars, respectively, while susceptible and resistant cultivars showed 33 common ASVs.

**Fig. 6:**
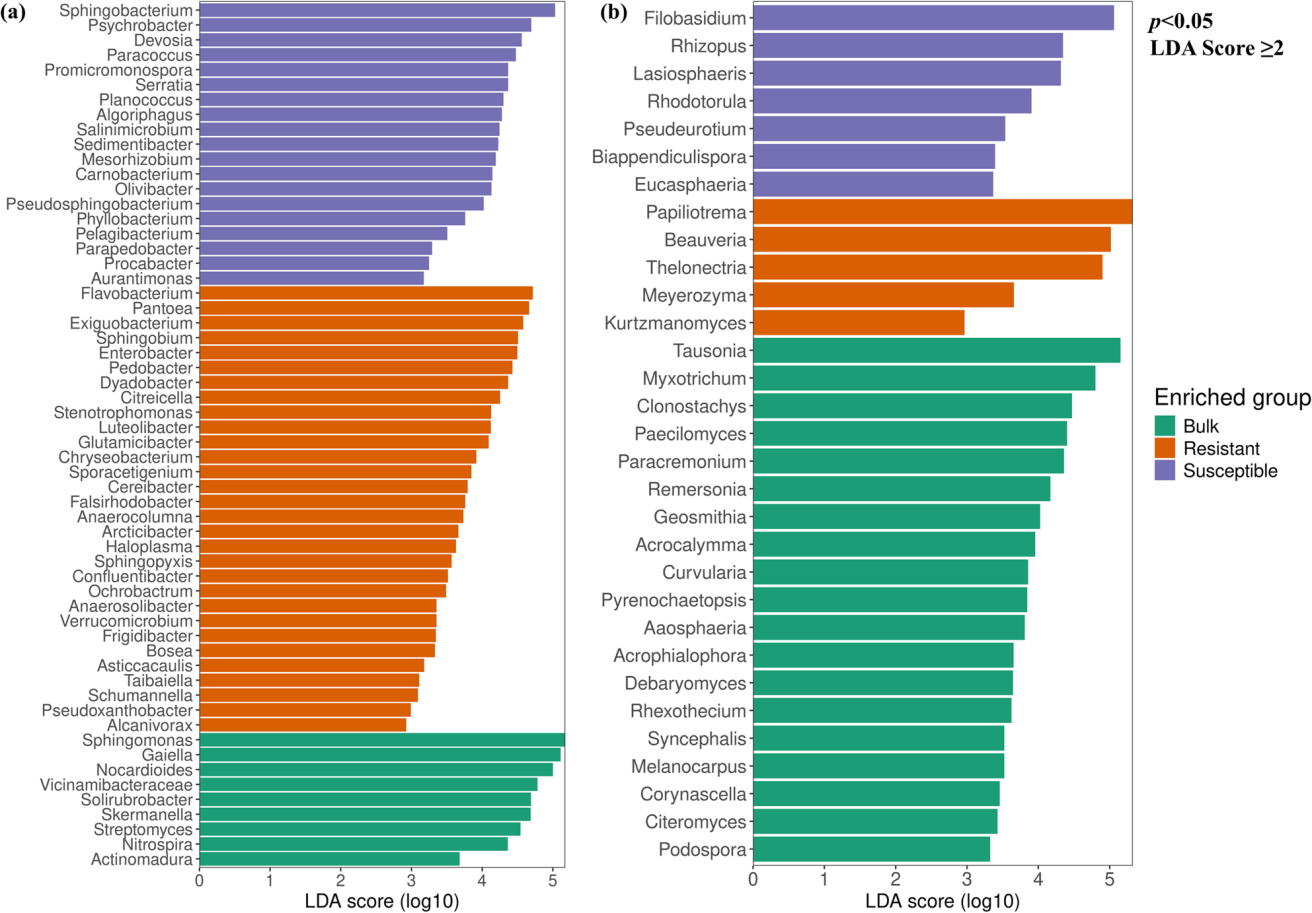
Histogram of the linear discriminant analysis (LDA) scores, where the LDA score indicates the effective size of significantly enriched taxa for bacterial (**a**) and fungal (**b**) populations

**Fig. 7:**
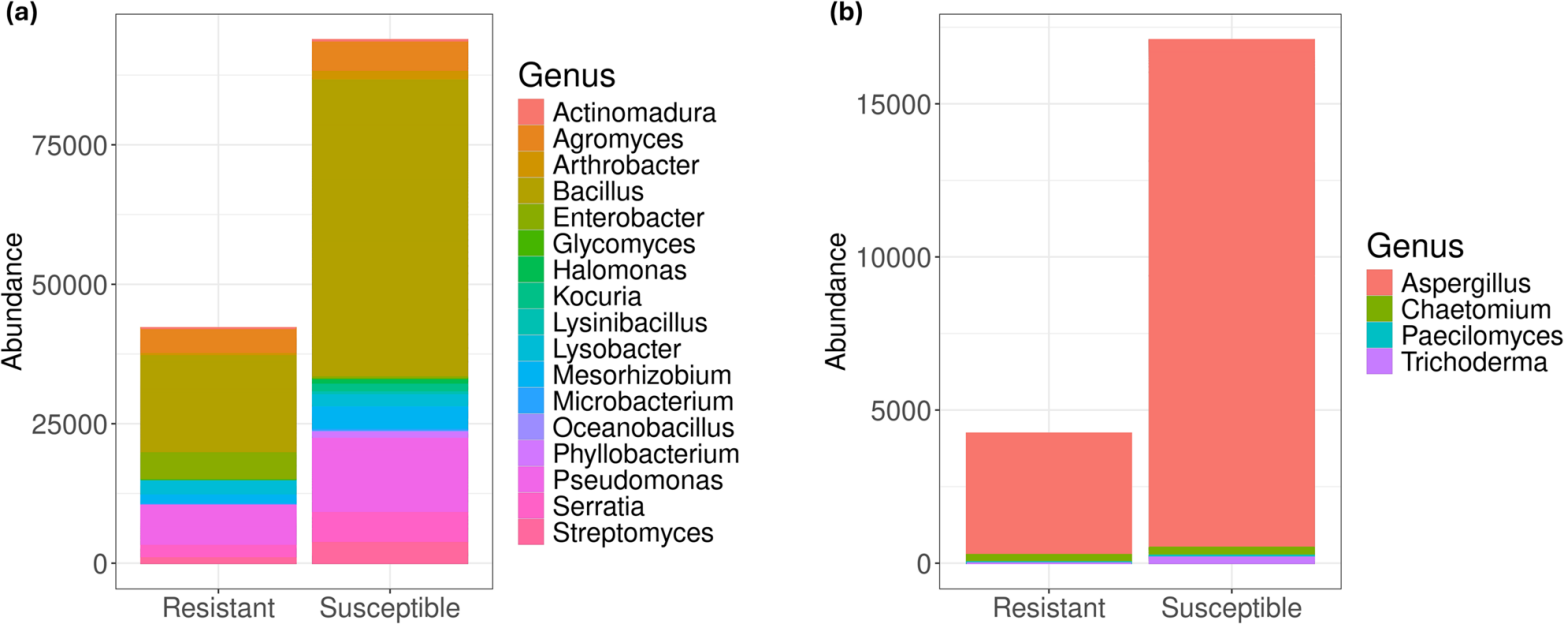
Abundance of (**a**) bacterial and (**b**) fungal beneficial genera in resistant and susceptible cultivars

To gain deeper insights into the differences in community structure, we analysed the bacterial and fungal taxa that were significantly enriched or depleted. Discriminant function analysis (*p* < 0.05, LDA score ≥ 2) revealed that 242 bacterial genera and 73 fungal genera (Table S3) were key contributors to the observed community distinctions. There were 180 bacterial and 57 fungal genera significantly enriched in bulk samples, 33 bacterial and seven fungal genera enriched in the resistant cultivar, and 29 bacterial and nine fungal genera enriched in the susceptible cultivar. Among the 180 bacterial taxa that were enriched in bulk samples, *Sphingomonas*, *Gaiella*, *Nocardioides*, *Vicinamibacteraceae*, *Candidatus*, and *Nitrocosmicus* were among the most represented genera. In the resistant cultivar, *Acinetobacter*, *Flavobacterium*, *Azotobacter*, *Sphingobium*, and *Pantoea* were dominant, whereas the susceptible cultivar was dominated by *Sphingobacterium*, *Pseudomonas*, *Devosia*, *Psychrobacter*, and *Promicromonospora* (Fig. 6a, Table S3). The fungal community was differentially enriched with *Tausonia*,* Aspergillus*,* Myxotrichum*,* Beauveria*, and *Cladosporium* in bulk samples, *Ceratocystidaceae_gen_Incertae_sedis*, *Plectosphaerella*, *Papiliotrema*, *Thelonectria*, and *Pezizomycotina_gen_Incertae_sedis* in the resistant cultivar, while the susceptible cultivar were dominated by *Alternaria*, *Filobasidium*, *Hypocreales_gen_Incertae_sedis*, *Lasiosphaeris* and *Rhizopus* (Fig. 6b, Table S3). The results also showed that the susceptible cultivar had a significantly higher abundance of several beneficial bacterial genera such as *Lysinibacillus*, *Actinomadura*, *Halomonas*, *Kocuria*, and *Phyllobacterium*. This observation led to a comparison of the abundance of several other beneficial genera between the two cultivars, although the difference was not statistically significant (*p > *0.05, Figure S1-S2). The comparative analysis also revealed a higher distribution of beneficial genera in the susceptible cultivar and these genera included *Bacillus*, *Streptomyces*,* and Trichoderma* (Fig. 7).

### Microbial co-occurrence network analysis in bulk, resistant, and suceptible samples

**Fig. 8:**
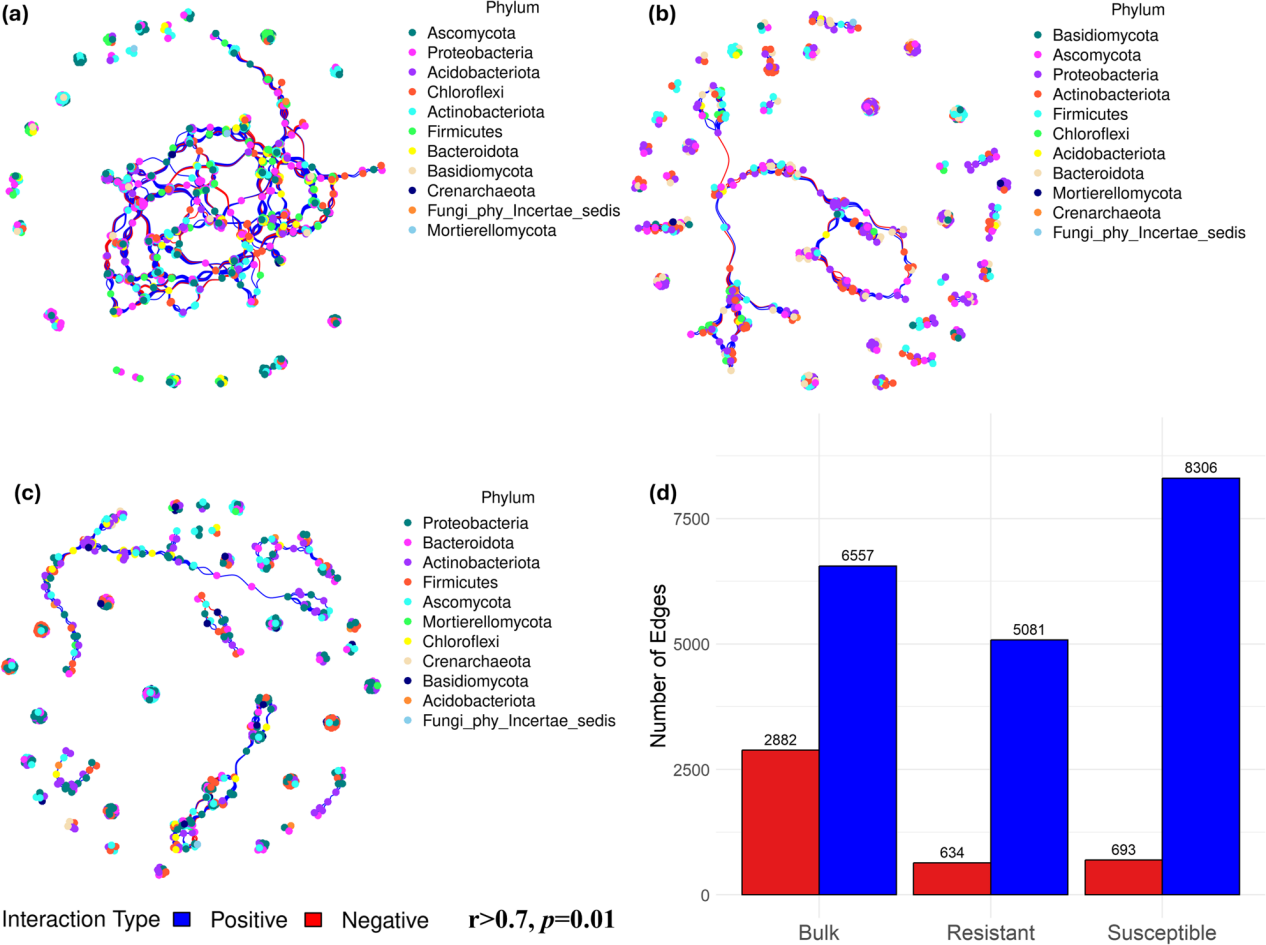
Co-occurrence network of bacterial and fungal populations in (**a**) bulk, (**b**) resistant, and (**c**) susceptible cultivars with (**d**) showing a comparison of positive and negative interaction in the network

Co-occurrence networks were constructed based on significant correlations among the microbial populations (Spearman correlation coefficient *r* > 0.7, *p* < 0.01) to explore the interconnections between the different taxa. As shown in Fig. 8 and Table S4, the network topology varied for each group of samples. The highest number of connections was observed in bulk soils (9439), followed by susceptible (8999) and resistant (5715) cultivars. The average path length values were lower in resistant and susceptible cultivars (8.75 and 9.37, respectively) than in bulk soils (14.89, Table S4). Moreover, the microbial community in the susceptible cultivar formed more positive interactions compared to bulk and resistant cultivar network. It also harboured a higher number of negative interactions compared to the resistant cultivar (Fig. 8, Table S4). We also noted that the clustering coefficient was higher in the susceptible cultivar (0.99) than in the resistant cultivar (0.97) and bulk samples (0.81) (Table S4). Taxonomy annotation showed that the microbial network of the bulk soil was dominated by Ascomycota, Proteobacteria, Acidobacteriota, and Chloroflexi (Fig. 8a). The resistant cultivar were dominated by Basidomycota, Ascomycota, Proteobacteria, and Actinobacteriota (Fig. 8b), while the susceptible cultivar was dominated by Proteobacteria, Bacteriota, Actinobacteriota, and Firmicutes (Fig. 8c).

### Foa presence in the soil and in vitro antagonistic assay

Foa presence in the root surrounding soil was evaluated using specific primers that amplify the insertion site of the *Fot1* transposable element, which is specific to Foa isolates. The results showed positive amplification of this segment, as shown in Figure S2 and confirm the presence of the pathogen in the soil. In the in vitro antagonistic assay, we identified seven bacteria that effectively inhibited the growth of Foa (Figure S4). The percentage of inhibition ranged from 65.55 to 78.14% (Table 1). The taxonomic identification of the seven isolates showed that they all belong to the *Bacillus* genera and are closely related to *B. mojavensis*,* B. halotolerans*, *B. siamensis* and *B. velezensis* (Table S5).

**Table 1:** Percentage inhibition of antagonistic bacteria against Foa

Isolate	ZJH1	ZJH2	ZJH3	ZJH4	ZJH5	ZJH6	ZJH7
%Inhibition	78.14 ± 2.31a	77.03 ± 2.31ab	72.96 ± 1.28bc	76.66 ± 1.92ab	70.37 ± 0.64c	62.22 ± 2.22d	68.67 ± 0.0c

## Discussion

The Moroccan oasis is home to different date palm cultivars, among which Black Bousthammi and Jihel are predominant in the Zagora oasis. While Black Bousthammi shows a strong resistance to Foa, Jihel cultivar is highly affected [26]. The main objective of this work was to decipher the bacterial and fungal communities associated to the roots of these two cultivars and explore the influence of each on the microbial community assembly. Furthermore, given that the rhizosphere microbiome can constitute the first line of defense against soil-borne pathogens [39] and trigger disease resistance [40], we explored the abundance and distribution of beneficial micro-organisms, that could be antagonistic to Foa, in the two cultivars. As the analysis of environmental variables showed no significant differences (*p* > 0.05), we assumed that the variations in the microbial community were only influenced by the cultivar genotype.

Research has shown that plants attract rhizosphere microbes from the bulk soil [41, 42]. Consequently, the microbial community in the rhizosphere consists of microbes from bulk soil, albeit in varying proportions. This means that some microbes are more abundant in the root compartment than in bulk soils, whereas others are less represented. For example, the phyla Actinobacteriota, Chloroflexi, and Acidobacteriota, which were prevalent in the bulk soil, were less abundant in the roots of the two date palm cultivars, in contrast to the increased abundance of Proteobacteria, Firmicutes, and Bacteroidota. In comparison with other studies on the bacterial population associated to date palm roots [10, 14, 16, 19], the results reported here displayed considerable similarities in the represented phyla, with the primary variation stemming from differences in their relative abundance. For instance, the high abundance of Proteobacteria reported in the present analysis was concordant with previous studies from Tunisia [14] and Oman [19]; however, in other studies, the bacterial population was dominated by the Actinobacteriota phylum [10, 16]. In addition, compared to these studies, the present analyses identified more bacterial phyla associated with date palm roots, indicating a higher bacterial diversity in the Moroccan oasis. The analysis of the fungal community identified 13 distinct fungal phyla associated to date palm roots, dominated by Ascomycota and Basidiomycota. This observation aligns with previous research that documented the prominence of Ascomycota and Basidiomycota populations in the roots of date palm as reported by Ferreira et al. [10] and Yaish et al. [43]. Additionally, it is worth noting that the present experiment uncovered a greater diversity of fungal phyla than what was reported in the aforementioned studies, confirming the observation from in the bacterial community analysis.

The described results demonstrated a clear distinction in microbial composition between the bulk soil and root compartments, reinforcing the idea that microbial communities are specifically selected in the root zone. Moreover, PERMANOVA results indicated a significant difference in community structure between the two genotypes, with a more pronounced divergence observed in bacterial populations (*p* = 0.017) compared to fungal populations (*p* = 0.131). This finding contrasts with the observations of Ferreira et al. [10], who reported that the fungal community exhibited greater sensitivity to variations in date palm cultivars than the bacterial community. This divergence in results highlights the complex and potentially nuanced interactions between microbial communities and plant genotypes. We also delved into genera that exhibited significant differences in abundance between the bulk soil, resistant and susceptible cultivar samples, to identify the key populations that contribute to the community differences observed across these three compartments. Interestingly, the susceptible cultivar harbored higher abundances of *Pseudomonas*, *Bacillus*, *Streptomyces*,* Lysinibacillus*, *Actinomadura*, *Halomonas*, *Kocuria*,* Phyllobacterium*,* Serratia*,* Oceanobacillus*,* Aspergillus* and Trichoderma. These beneficial genera play vital roles in the management of plant fungal diseases [44–53], and several of them such as *Pseudomonas*, *Bacillus*, *Streptomyces* and *Tricoderma*, showed promising results in the management of bayoud disease [54–58]. This enrichment might be the result of pathogen pressure, triggering the recruitment of beneficial microbes in the rhizosphere. This strategy, called “cry-for-help” [59], is activated by plants under pathogen infection through the release of various compounds in the surrounding environment. This is followed by the assembly of beneficial microbes that can hamper and limit pathogen infections [60]. This strategy is broadly represented among different plants undergoing pathogen attacks. For instance, a comparative metatranscriptomics study between disease-suppressive and non-suppressive soils reported a higher abundance of biocontrol agents (BCAs) such as *Arthrobacter* spp. and *Pseudomonas* spp. in disease conductive soil for *Rhizoctonia solani* [61]. Moreover, gene expression patterns revealed a higher presence of antibiotic genes in non-suppressive soils [61]. The enrichment of BCAs in a cucumber variety that showed susceptibility to *F*. *oxysporum* f.sp. *cucumerinum* was also reported in microbial community analysis compared to a variety that showed resistance [62]. These observations are in accordance with the reported findings and further highlight the important role of the plant microbiome in mitigating pathogen infection. 

## Conclusion

This study aimed principally to compare the microbiome of two date palm cultivars, one resistant and the other susceptible to bayoud disease. As revealed by PERMANOVA, the two cultivars harboured significant differences in their root-associated microbial communities. Discriminant function analysis showed that the susceptible cultivar exhibited a significant enrichment of beneficial genera such as *Pseudomonas*,* Lysinibacillus*, *Actinomadura*, *Serratia*, *Halomonas*, *Kocuria*, and *Phyllobacterium*. A deeper comparative abundance analysis of other beneficial genera, which was not statistically significant, also exhibited a higher abundace in the suceptible cultivar. These beneficial genera encompassed *Bacillus*, *Streptomyces*,* and Trichoderma.* This higher abundance of beneficial genera suggests a pathogen-driven recruitment strategy, and the isolation of antagonistic bacteria showed that the susceptible cultivar was effectively associated with bacteria producing antifungal compounds. Future assays incorporating *in planta* experiments should be considered for the functional validation of the biocontrol potential of these isolates. Nonetheless, this study represents the first effort to explore the differences in microbiomes between bayoud-resistant and susceptible date palm cultivars in Morocco and provides a fundamental step toward integrating microbiome research into the sustainable management strategies for bayoud disease.

## Supplementary Information

Supplementary Material 1.

Supplementary Material 2.

## Data Availability

The 16 S rRNA and ITS gene sequencing data and associated metadata were deposited to NCBI SRA repository under BioProject [PRJNA1209931] (https://www.ncbi.nlm.nih.gov/bioproject/PRJNA1209931).

## Abbreviations

Foa: Fusarium oxysporum f. sp. albedinis
BCA: Biocontrol agent
PBS: Phosphate-buffered saline
ASV: Amplicon sequence variants
PCoA: Principal coordinate Analysis
db-RDA: Distance-based redundancy analysis
LDA: Linear discriminant analysis
LEfSe: Linear discriminant analysis Effect Size
PERMANOVA: Permutational Multivariate Analysis of Variance
