# Supplementary material for: Comparative metagenomic analysis of bacterial and fungal communities associated with bayoud-resistant and susceptible date palm cultivars in the Zagora oasis-Morocco
Source: BMC Microbiol. 2026 Feb 23;26:287. doi: 10.1186/s12866-026-04837-8 (PMC13037060; doi:10.1186/s12866-026-04837-8)
Supplement: Supplementary file 1 — Supplementary Material 1. [file 12866_2026_4837_MOESM1_ESM.docx]

Supplementary Figures


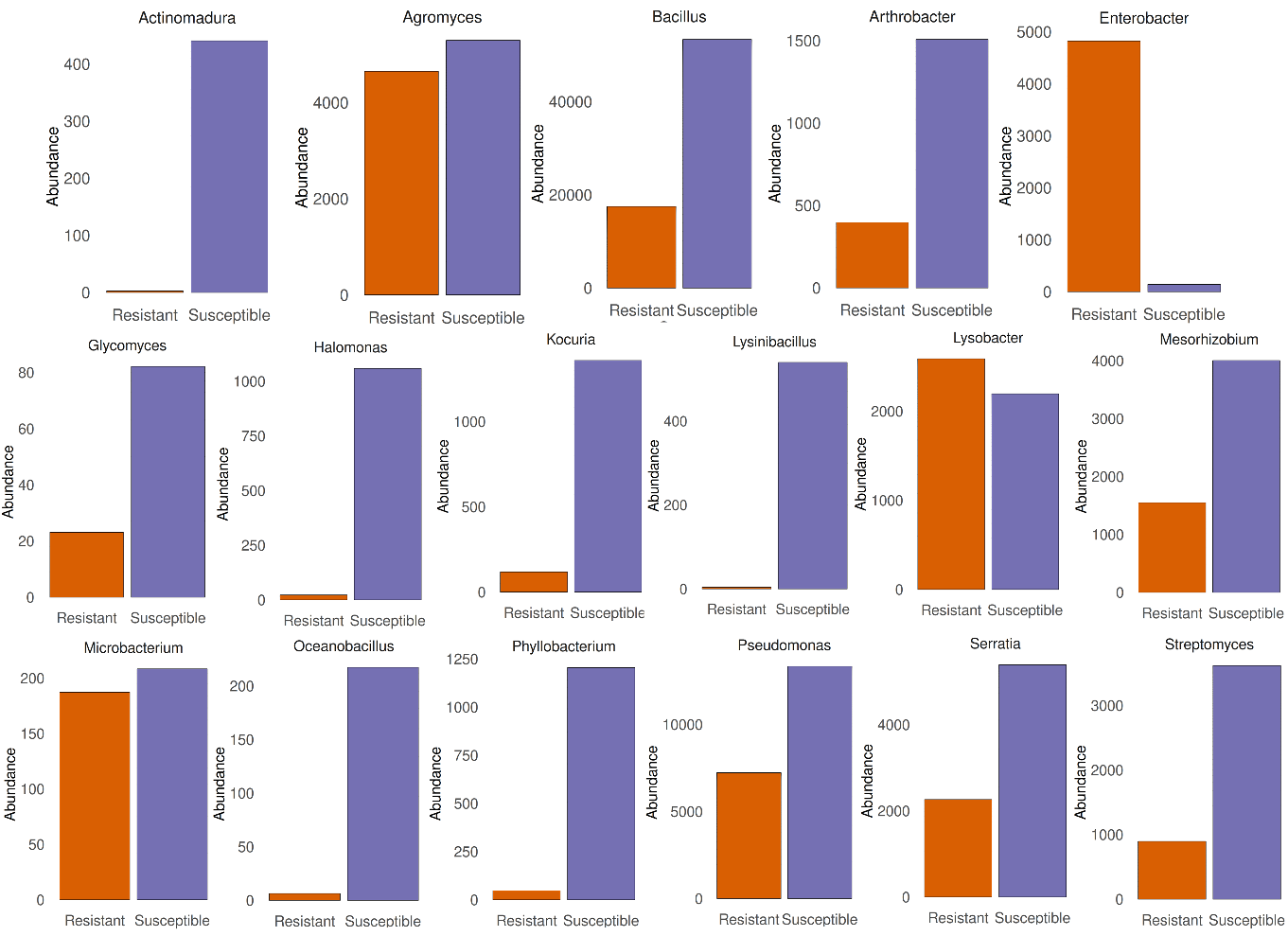


Figure S1: Comparative abundance of several beneficial bacterial taxa between resistant and susceptible cultivars


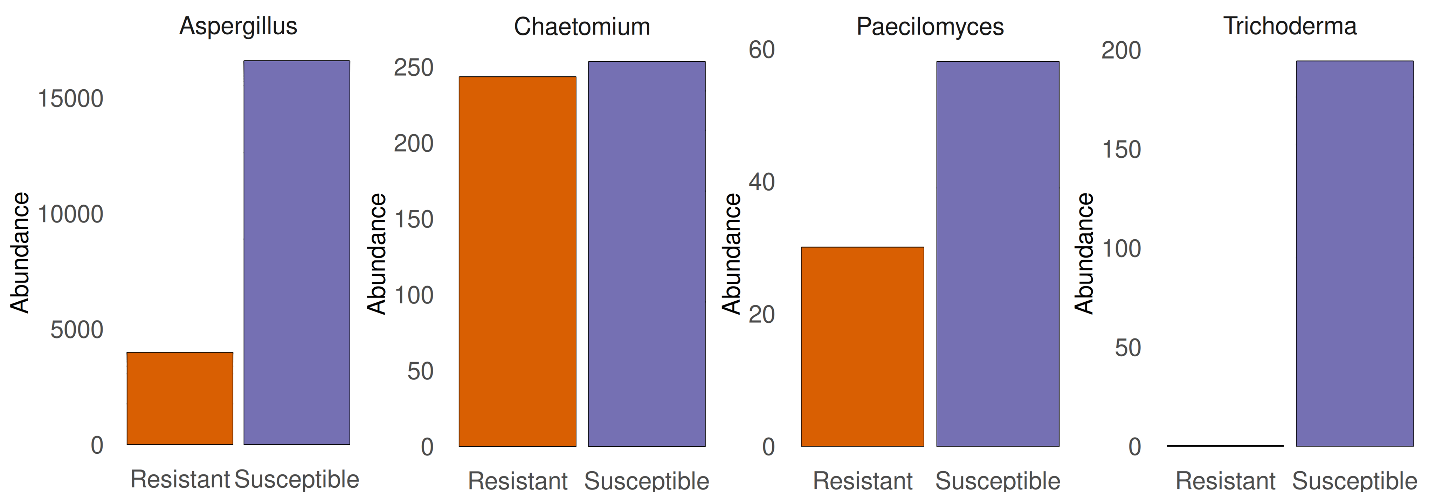


Figure S2: Comparative abundance of several beneficial fungal taxa between resistant and susceptible cultivars


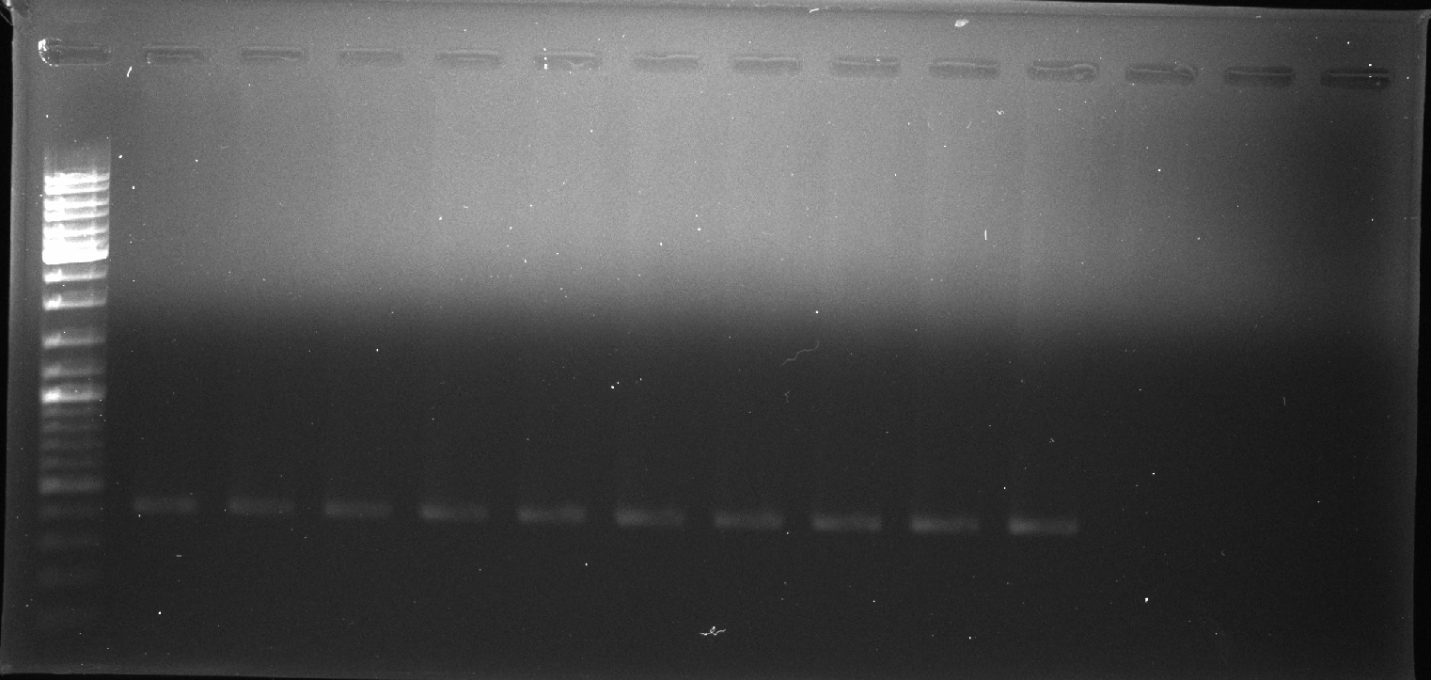


Figure S3: PCR detection of Foa using TL3 and FOA28 primers


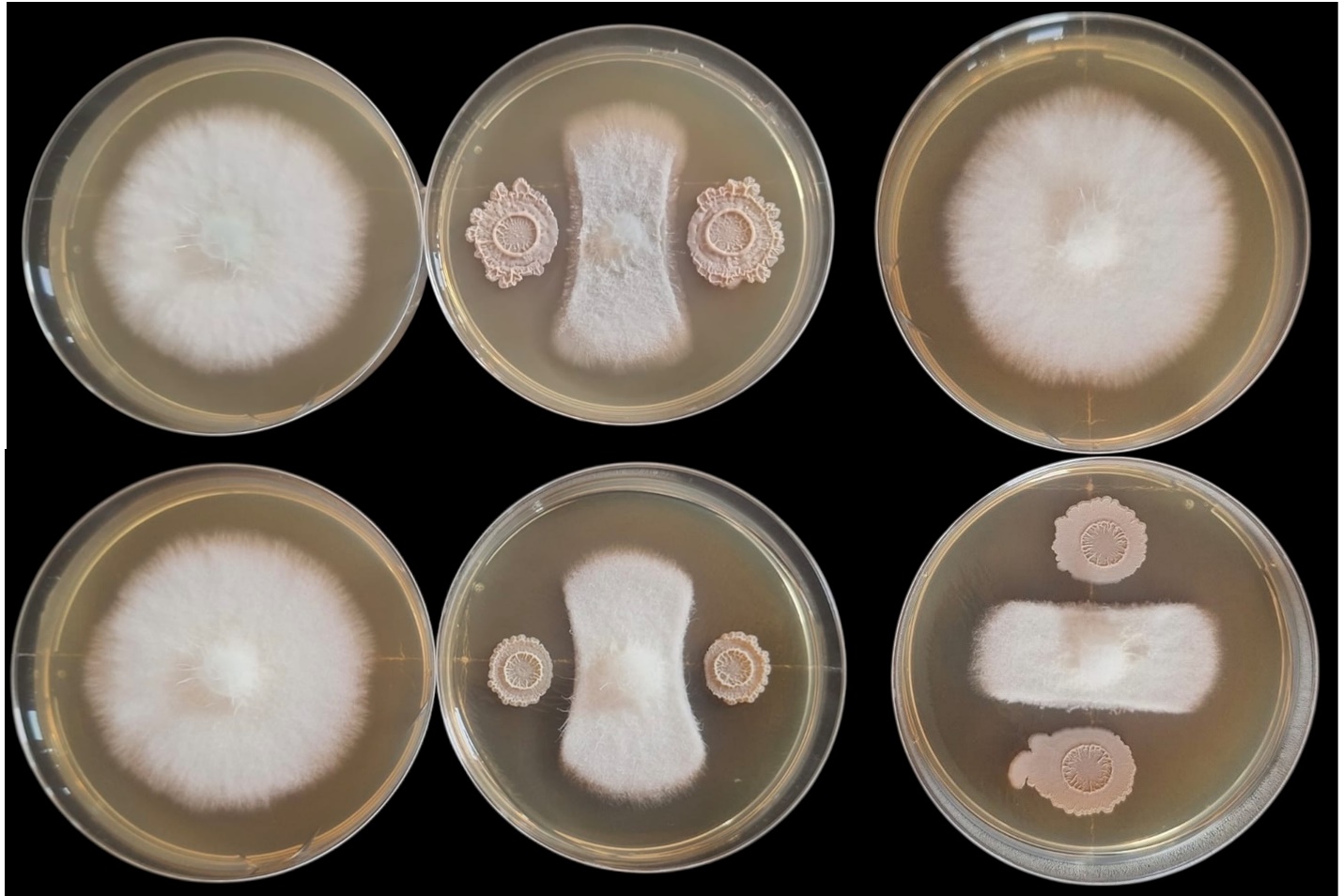


Figure S4: Illustration of in vitro antagonistic activity against Foa
